# Understanding and Addressing Upcoding

**DOI:** 10.1111/1475-6773.14606

**Published:** 2025-03-17

**Authors:** Bryan Dowd

**Affiliations:** ^1^ Division of Health Policy and Management University of Minnesota Minneapolis Minnesota USA

## 1 Introduction

The term “upcoding” has several interpretations. In a recent scoping review, the RAND Corporation defines upcoding as, “…the coding of a patient to a higher complexity level than they would be if payment were unrelated to complexity.” [1] In some contexts, upcoding implies fraudulent behavior. For example, Coustasse defines upcoding as submission of payment codes for more severe and expensive diagnoses or procedures than the provider actually diagnosed or performed [2]. The Centers for Medicare and Medicaid Services (CMS) provides the following example of upcoding: billing a follow‐up visit using a higher‐level evaluation and management code, such as a comprehensive new‐patient office visit [3].

Coding diagnoses for medical conditions that the patient never had certainly constitutes fraud, but CMS already allows coding additional diagnoses that increase the cost of treating a patient for a specific condition. The adjustment is made through interaction terms among diagnoses in the computation of risk scores. For example, capitation payment for a diabetic patient is higher if the patient also has congestive heart failure [4]. The following analysis assumes there is universal agreement that fraudulent upcoding is rightly illegal and focuses on a suggestion to improve the accuracy of legal upcoding.

There is an adage, “Never assume malevolence when stupidity works just as well.” A modification might read, “Never assume malevolence when Econ 101 works just as well.” A useful guide to understanding human behavior is that people generally do what they are paid to do. In traditional fee‐for‐service (FFS) Medicare (TM), health care providers are paid to provide services to patients. In contrast, MA plans are paid a capitation amount, and the capitation amount is higher for patients with more medical conditions. Thus, MA plans are rewarded for coding more diagnoses. If we rule out fraudulent coding then the additional diagnoses coded by the MA plan compared to a similar patient in TM represent diagnoses that are observed and documented, but not necessarily diagnoses that increased the cost of treating the patient over the time period used to compute the capitation payment. CMS understands the problem and has responded by reducing the capitation payments to MA plans by 5.9%–an amount that CMS is required to apply, but less than CMS is authorized to use [5]. This uniform adjustment has been criticized, because upcoding varies from MA one plan to another [6].

## 2 Upcoding Extends Beyond MA Plans

Upcoding is far from an MA‐only problem, although MA accounts for the greatest economic impact of upcoding [7]. In its Accountable Care Organization (ACO) alternative payment models, CMS pays ACOs on a FFS basis, but ACOs are subject to rewards and penalties based on their performance relative to an administratively determined benchmark. The benchmark, in turn, is computed using diagnosis‐based risk adjustment. The more diagnoses that are coded for the patient, the higher the benchmark, and the greater the probability that the ACO will be rewarded with shared savings, rather than penalized. As a result, ACOs that face the risk of a financial penalty for spending above the benchmark have a similar incentive to upcode as MA plans, and they respond accordingly [8]. In response, CMS has found it necessary to impose upcoding ceilings on ACOs in TM, similar to those imposed on MA plans. A recent study also found upcoding by hospitals, whose prospective payments are a function of diagnosis‐based risk payments [9].

Upcoding is symptomatic of larger issues in provider payments. Should providers be paid for services or for diagnoses? FFS payments or risk‐adjusted capitation? Each payment system has advantages and disadvantages. FFS payment provides a claims‐based record of what actually was done to the patient, but FFS payment also encourages the prescription of low‐value and wasteful services [10]. Capitation payment, adjusted for the enrollee's diagnosis‐based “risk” discourages overuse, but encourages upcoding and skimping on services unless health outcomes are closely monitored.

## 3 Approaches to Upcoding

To balance these incentives, Newhouse proposed a blended payment system [11]. CMS's efforts to improve risk adjustment in its V28 initiative involve eliminating some of its Hierarchical Condition Categories (HCCs), and altering the mapping of diagnoses into the remaining HCCs [12]. Lieberman and Ginsburg [6] and Jung, Carlin, and Feldman [13] propose upcoding adjustments that are tied to the MA plan's observed level of upcoding. Other proposals include limiting the sources of diagnosis codes and developing diagnosis weights specific to the MA sector [13].

Another approach to setting capitation rates is to link the allowable diagnoses to those for which the patient received treatment or those that directly affect the cost of that treatment. Here is one suggestion:

1. Put some teeth in the primary diagnosis. All payment computations would begin with a primary diagnosis for each patient encounter—the condition for which the patient was treated during the encounter (primary diagnosis).
2. Other diagnoses could be coded for the encounter, but additional payment would be made only for the primary diagnosis or for a *CMS‐certified* interactive diagnosis [4] that added to the cost of treating the primary diagnosis for at least one encounter. Plans would not be paid for diagnoses that simply were observed or recorded. Jung, Feldman, and Carlin found that 36.1% to 46.6% of the risk scores collected from health risk assessments and chart reviews were not associated with increased use of resources [13].
3. As is currently the case with the −25 modifier, there could be more than one primary condition for a single encounter if the patient is treated for multiple conditions during a single encounter, but if the medical records are audited, they should show the treatment(s) and procedure(s) that were provided for each primary diagnosis at each encounter.
4. Diagnoses coded according to the rules above would be the diagnoses used to compute the capitation payments for MA plans and other capitated entities. Capitation would continue to be prospective, with the coming year's payment based on the previous years' data.

This approach will not solve all the problems of the Medicare program. For example, outlays will still exceed revenue [14], and TM's level of resource use and fee schedule will continue to set the payment rate for diagnoses in the capitation system [13, 15]. Any payment system will require monitoring and some further diagnosis interactions may need to be added to CMS's current list. But MedPAC estimates that losses to upcoding reached $50 billion in 2024 [5], and thus a sizable investment in addressing the issue is justified.

## Conflicts of Interest

The author declares no conflicts of interest.

## Data Availability

The data that support the findings of this study are found in the references.
